# Realising potential: Strong leadership and resilience of Pacific emergency care clinicians

**DOI:** 10.1016/j.lanwpc.2022.100583

**Published:** 2022-08-31

**Authors:** The Lancet Regional Health – Western Pacific

The Pacific Island Countries and Territories (PICTs) are home to more than 12 million people, dispersed across 22 countries in the world's largest ocean. Although great strides in the pursuit of health have been made in the PICTs, the region remains vulnerable to the health impacts of climate change, natural disasters, and disease outbreaks. As such, strengthening emergency preparedness and response is a regional health priority. An effective emergency care system is central to preparing for and responding to health emergencies. When emergency care systems become paralysed or collapse entirely, both direct and indirect mortality increase considerably. An estimated 54% of deaths in low-income and middle-income countries (LMICs) could be treated by improved emergency care accessibility and quality.

In this issue of *The Lancet Regional Health – Western Pacific*, the Pacific Emergency Care Series provides a rare insight into the experiences of emergency care health-care workers (HCWs) at the frontline of the COVID-19 pandemic in Pacific LMICs. The series of qualitative studies conducted via a collaboration between PICTs and Australian researchers show substantial gaps in emergency care capabilities and poor integration with health emergency response plans. It provides a blueprint for closing these gaps in emergency care preparedness and response to rapidly mobilise key personnel, material, and organisational resources to manage future health emergencies in the PICTs.

The duty to treat when one faces a considerably higher risk of infection and death was complicated at the outset by the lack of permanent and trained staff in emergency care facilities across many PICTs. HCWs in emergency care performed multiple roles early in the pandemic, leading many to experience distress and exhaustion, amplifying the physical, mental, and professional challenges posed by the pandemic. This was made worse by existing equipment and infrastructure constraints. There was a widespread lack of access to and availability of personal protective equipment (PPE), and some emergency care facilities had only one working ventilator in their intensive care unit. Resource-allocation decisions about which patients to treat were a dire reality faced by HCWs in the PICTs. These studies shed light on the critical ethical dilemmas that emergency care HCWs in the PICTs faced during the pandemic. An understanding of ethical issues that might arise in health emergencies, as well as clear ethical guidance, can help HCWs communicate with patients and their families about difficult health-care decisions and mitigate stress related to ethical dilemmas. As such, emergency care training for HCWs in the PICTs must incorporate ethics and mental health to break the vicious cycle of workload burnout and staff turnover during health emergencies.

Despite these challenges, HCWs showed resourcefulness and resilience by establishing new clinical spaces, modifying PPE, and adapting triage processes. For example, modifying the prioritisation and streaming of patients to isolate individuals with a high transmission risk and continue to provide care for patients with non-COVID-19 health conditions. This process was invaluable to minimise mortality and morbidity and additional burden on the health system. Strong leadership and governance played an important role in guiding the adaptation of crucial emergency care processes. HCWs assumed leadership roles in national committees and advocated for evidence-based care despite pandemic politics. They demonstrated their valour and expertise by guiding service provision, policy, and an integrated multisectoral approach to the pandemic. HCWs were also able to engage political support to bolster service provision and infrastructure.

The Pacific Emergency Care Series serves as an important guide to HCWs and policy makers for strengthening emergency care in LMICs. Communication and transparency to foster trust and support in new processes and enhance workplace culture were key to prevailing over fear and disunity caused by the pandemic. Moving from top-down health system governance and decision making to a more collaborative approach ensured that the views and experiences of HCWs in emergency care were incorporated to develop practical solutions to emergency care shortcomings. The wealth of expertise and leadership capabilities of HCWs in emergency care has revealed an invaluable avenue for strengthening emergency care globally. The resilience and unity shown by HCWs in emergency care in PICTs show what can be achieved now, and in the future, to strengthen emergency preparedness and response in the context of resource-limited health systems. In this context, learning becomes a two-way process, further strengthening connections and vital partnerships among HCWs, researchers, managers, and policy makers at the regional and global levels to support resilient and sustainable emergency care systems.

